# The great pretenders? Individuals’ responses to threats to their remote worker identities

**DOI:** 10.3389/fpsyg.2023.1224548

**Published:** 2023-11-06

**Authors:** Bruno Felix, Bruno Lorencini Tiussi, Jasmin Mahadevan, Rogério Correia Dias

**Affiliations:** ^1^Fucape Business School, Vitória, Brazil; ^2^Hochschule Pforzheim, Pforzheim, Germany

**Keywords:** remote work, identity, stigma, identity threat, coping responses

## Abstract

**Introduction:**

This study aims to understand (a) how remote workers respond to threats to their identity and (b) the conditions in which each coping response tends to occur more frequently.

**Methods:**

To this end, we pursued a grounded theory approach, conducting interviews with 71 individuals who chose to work remotely.

**Results:**

Our model and theoretical propositions create insights into how remote workers respond to negative stigma from a range of origins. While some responses lead to restructuring the remote workers’ identity (identity restructuring responses), others involve keeping the enactment of such identity (identity-preserving responses) or maintaining a paradoxical relationship between restructuring and preserving the identity (paradoxical identity work responses). We also theorise on the conditions under which each response is more likely to occur.

**Discussion:**

We expand the predominant focus on the meso and macro aspects of this type of work to the micro-interactions in which these individuals engage, thus highlighting how identity is made, performed, created, and enacted, within specific boundary conditions. In addition, by reflecting upon remote workers’ identity threats in light of the wider macro context. We also explore the conditions under which specific kinds of responses tend to emerge.

## Introduction

“They should pretend to work somewhere else” (Musk, 2022) –(Elon Musk talking about the future of remote workers at his company, Tesla).

The COVID-19 pandemic has forced individuals from many occupations to stay at home to avoid spreading the virus (Cooke et al., 2020). As a result, the world has seen unprecedented numbers of workers from a wide range of occupations working from home (Vaziri et al., 2020; Campbell and Gavett, 2021; Moss, 2021). After the most critical moment of the COVID-19 pandemic, some companies made their employees return to the offices, while others decided to take advantage of the know-how generated on how to work remotely and opted to maintain this type of work arrangement, either partially or totally (Guidetti et al., 2022).

The Tesla company, led by the entrepreneur and manager Elon Musk, is an example of an organisation that does not look upon remote work favourably and has decided to return to work in person. Days after Tesla announced that all its employees should return to work in the office full-time, Musk was asked about the company’s controversial decision, and the quote that introduced this article was his answer. His tweet hints at a belief that remote workers are not productive and only “pretend to work” and, furthermore, indicates that they would have no place at Tesla. Although his answer does not represent a homogeneous view of the labour market (Beckel and Fisher, 2022), it symbolises a recurring perception that some workers who choose to work remotely do so not because of a search for productivity but because they are not sufficiently dedicated or committed to work (PWC, 2018). Since this judgement can clearly harm the career and performance of remote workers (Barhate and Hirudayaraj, 2021), a question arises: how do remote workers respond to this negative judgement of their value as professionals?

Threats to remote workers’ self-concept are as ubiquitous as they are unsettling, in response to which a debate on the phenomenon has developed. For example, some studies emphasise the negative stigma surrounding remote work (Bornstein, 2013; Barhate and Hirudayaraj, 2021), while others explore the difficulties of creating and maintaining a positive workplace identity while working remotely in the face of this stigma (Barsness et al., 2005). There is also evidence that increased separation from other members of the organisation may diminish employees’ visibility, which increases the likelihood that they need to manage impressions about who they are (Bailey and Kurland, 2002) and reduces the opportunities to manage their identity successfully (Barsness et al., 2005). These studies share an emphasis on identity, which is the response that individuals offer when answering the question, “Who am I?” (Petriglieri, 2011), and how the negative judgements of significant others impact the experiences of remote workers.

In this study, we seek to expand this incipient connection between the remote work literature and remote workers’ identities by exploring a very relevant phenomenon: identity threats. This concept refers to “an instance in which a participant perceived that the non-territorial workspace impeded his or her ability to affirm or display an aspect of identity” (Elsbach, 2003, p. 632). Despite the extensive knowledge of the consequences that identity threats generate for the worker concerning authenticity (Felix, 2020), performance (Steele, 2020), willingness to support organisational decisions (Nag et al., 2007) and intention to remain (Trevor and Nyberg, 2008), there is still a need to better understand how individuals handle resistance to their remote workers’ identity. To expand the understanding of the negative stigma toward these individuals, this study aims to understand (a) how remote workers respond to threats to their identity and (b) the conditions in which each coping response tends to occur more frequently. To this end, we employed a grounded theory approach and conducted interviews with 71 individuals who chose to work remotely.

This study contributes to the theory about the identity of remote workers. We expand the predominant focus on the meso and macro aspects of this type of work (Lin, 2011; Adamovic, 2018; Mockaitis et al., 2018; Donnelly and Johns, 2021; Williams et al., 2021) to the micro-interactions in which these individuals engage, thus highlighting how identity is made, performed, created and enacted, within specific boundary conditions. In addition, by reflecting upon remote workers’ identity threats in light of the wider macro context—int his case: the political framing of remote workers’ identities in Brazil—we extend the analytical lens to the macro-level, thus pointing to the relevance of analysing how micro-interactions and macro-level boundary conditions inform each other. Thirdly, research on identity threats has traditionally framed attempts to minimise harm (Blanz et al., 1998; Breakwell, 2015). However, in this article, we follow the call of Roberts et al. (2009) for research that also explores the conditions under which specific kinds of responses tend to emerge. Given the growing trend in the number of remote workers in the world and the fact that the negative judgements of them have not ceased (Megan, 2022), the identity-based micro approach adopted in this study proves to be an adequate perspective for making theoretical advances on the topic.

## Literature review

### Remote workers and stigma: what do we know thus far?

The managerial and academic interest in the topic of remote work is not a recent phenomenon (Tietze and Nadin, 2011), but it has grown intensely since the beginning of the COVID-19 pandemic (Brynjolfsson et al., 2020; Raišienė et al., 2020; Carsten et al., 2022). Studies have sought to explore issues such as the role of human resource management (Adamovic, 2018; Donnelly and Johns, 2021; Williams et al., 2021) and technologies and structures (Lin, 2011) in facilitating leadership to achieve effectiveness in remote work (Mockaitis et al., 2018). Studies that emphasise individual aspects tend to explore the effects of remote work on variables associated with worker productivity (Galanti et al., 2021). However, some have specifically explored the stigmatisation of these workers, as they are more susceptible to bias and at risk of discrimination (Bornstein, 2013). In this topic, we map studies that have specifically addressed this topic.

A topic that has often been addressed in the literature on the stigma suffered by remote workers is their need to manage the impressions that other people have of them. Previous studies suggest that when in-person supervision and face-to-face interaction are replaced by technology-mediated interactions, employees’ organisational visibility decreases, which enhances their need to manage other people’s impressions of them (Barsness et al., 2005; Shockley et al., 2021). Many employees today work from home, in satellite offices, from work centres, and on the road, and as a consequence, their dedication to work is less easily “seen” (Bailey and Kurland, 2002; Fang et al., 2022). Thus, remote workers may tend to adopt more impression management behaviours than their non-remote colleagues (Barsness et al., 2005; Bolduan et al., 2021), as they need to compensate for this limited natural visibility with some other strategic ways to make themselves visible.

Another topic that deserves attention in the literature refers specifically to the impacts that the negative stigma directed toward remote workers generates on their careers (Chung, 2020; Padavic et al., 2020; Becker et al., 2022; de Laat, 2022). This stigma reflects deep cultural assumptions that work requires and deserves undivided and intensive loyalty (Carnevale and Hatak, 2020; Byrd, 2022). Thus, remote work can be interpreted by superiors, co-workers and even the employee him or herself as a sign that the worker is violating these cultural assumptions (Williams et al., 2013; Cech and Blair-Loy, 2014; Barhate and Hirudayaraj, 2021). As a result, even though remote workers often work long hours, they may not be recognised and rewarded for such efforts and may not be considered for job assignments (Bailey and Kurland, 1999; Golden and Eddleston, 2020).

Although these studies are important for pointing out how remote workers seek to minimise the stigmas associated with them, as well as the effects of such stigmas, they are still limited in terms of understanding how employees deal with this threat to their self-concepts. We seek to advance this discussion inspired by sensitising concepts from the identity literature, which we present below.

### Identity

This study uses social identity theory (Tajfel and Turner, 1979) as a sensitising approach. According to this theory, an identity (self) is a definition that an individual builds about him or herself and that is constructed based on social interactions while searching to answer the question “Who am I?” or “Who are we?” (Yip et al., 2020; Felix et al., 2023c). Applying this concept, it is possible to suggest that a remote worker identity is not only related to working remotely but also an aspect of one’s life from which an employee defines him or herself. Identities have three main roles: they provide an individual with self-esteem (they lead to the creation of self-value), they are multiple (an individual does not see him or herself based on a single self-definition but rather in a few different ways), and they are dynamic (they can change over time) (Felix, 2020). In the following, we explain these properties in detail.

First, identities are reflections of how individuals interpret who they are; this self-representation is usually constructed to satisfy the necessity of seeing oneself in a positive light (Hennekam et al., 2021; Felix et al., 2023a). The self allows individuals to construct themselves cognitively and emotionally, which is very important for finding the security that is required to perform well at work (Dutton et al., 2010; Felix et al., 2023f). Therefore, a remote worker identity tends to be built by individuals who see the act of working from places other than the company’s official workplace as something interesting to the point of seeing themselves positively and feeling good about who they are by including this dimension in their self-concept.

Second, identities can coexist and conflict; they are not monolithic (Felix and Bento, 2018). Individuals build alternative identities and enact them based on how they see the interpretation of significant others and on other situational aspects that are involved in a given social context (Felix et al., 2018; Felix and Cavazotte, 2019; Wittman, 2019). Thus, seeing oneself as a remote worker does not mean that the individual cannot also see him or herself as someone who works in a corporate office.

Third, even though identities provide individuals with emotional and cognitive stability, they are dynamic (Conroy and O’Leary-Kelly, 2014; Felix et al., 2023c). Since the self reflects society and an individual’s immediate and broader context (Mead, 1934) and both can change over time, identities can also undergo changes during an individual’s life. Consequently, we suggest that when an individual constructs a remote worker identity, it does not mean that he or she cannot change his or her self-concept in terms of where he or she works. Next, we explain one of the explanations for why individuals alter their self-concepts: an identity threat.

### Identity threats

Identity threats are “experiences appraised as indicating potential harm to the value, meanings, or enactment of an identity” (Petriglieri, 2011, p. 644). One of the aspects that motivates the construction of identity is the promotion of self-esteem (Dutton et al., 2010). Thus, when individuals see themselves as remote workers, they do so because this identity helps them to see themselves positively. However, when significant others critique the intentions and productivity of remote workers (McDonald et al., 2022), these individuals may interpret this as an identity threat.

Identity threats usually originate from the actions of the person who does not accept a self-defining element of another individual; the threat is not situated in the person who occupies the threatening position but in the individual whose identity is threatened (Verkuyten et al., 2019). For an identity threat to be characterised, the threatened individual needs to signify a given interpersonal situation as harmful to his or her sense of self (Kang and Kim, 2022). This means that the same social encounter can be regarded as positive or neutral by one individual and as an identity threat by someone else (Leavitt and Sluss, 2015; Felix et al., 2023d).

**FIGURE 1:**
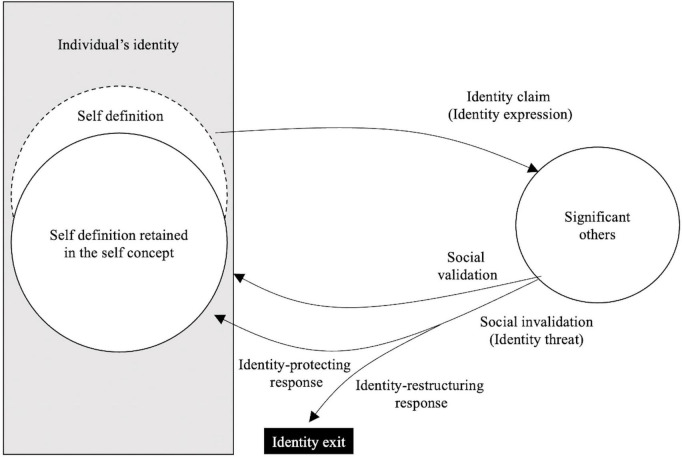
Summary of the literature review (Felix et al., 2023e).

When individuals are challenged by identity threats, their emotional and cognitive stability are challenged (Smith, 1991, Breakwell, 2015). However, this situation is typically temporary, as individuals tend to adopt coping responses that bring them back to the stability that is necessary to perform well at work (Branscombe and Ellemers, 1998). The literature highlights two main anticipated coping responses: identity-protecting and identity-restructuring responses (Piening et al., 2020). While the first is focussed on the source of the threat and does not change the threatened identity, the second re-signifies an element of the threatened identity to reorganise the individual’s sense of self-worth (Ashforth, 2001). Figure 1 summarises the literature review that we presented here.

After reviewing the literature on stigma toward remote workers and identities and presenting the sensitising concepts that support this study, we proceed to the methodological procedures.

### Research context

During the COVID-19 pandemic, Brazil has witnessed a clear division of opinions regarding the “stay-at-home” measures and, consequently, remote work. The negative portrayal of remote work by former president’s Jair Bolsonaro, who held the office during the time of research, contributed to the formation of a cultural bias against it, leading to increased stigmatisation of those who could work from home (Barros and Francisco, 2021). Despite most Brazilians agreeing with the adoption of social distancing measures to combat the pandemic, some segments of the population, mainly supporters of Bolsonaro, opposed the measures and downplayed the severity of the pandemic. This situation worsened during the pandemic, with many Brazilians feeling pressured to continue working in crowded offices or risk losing their jobs. According to data from the Brazilian Institute of Geography and Statistics, in 2020, only 22% of Brazilian workers could work from home, most of whom were from high-income households (IBGE, 2021). The political dimension of the issue turned the remote work debate into a divisive topic, with different segments of society taking opposing positions based on their political beliefs.

The negative stigma attached to remote work reinforces social and economic inequalities, as low-income workers often do not have the option to work from home, and they are more exposed to the risk of infection. The pandemic has exacerbated this situation, as many low-income workers are in essential sectors, such as healthcare and transportation, and have been unable to work remotely. According to a survey conducted by the Brazilian Institute of Public Opinion and Statistics, in March 2021, 79% of Brazilians agreed with the adoption of social distancing measures to combat the pandemic, while 17% disagreed (Ibope, 2021). However, Bolsonaro’s negative portrayal of remote work contributed to forming a cultural bias against it, leading to increased stigmatisation of those who could work from home (Fernandes, 2020). This shows that the theme of remote work has become a complex issue influenced by political positioning, with the potential to widen social and economic inequalities.

## Materials and methods

We conducted exploratory research that was carried out based on grounded theory procedures (Charmaz, 2014). This method was chosen because the lack of previous studies focussed on theorising about how remote workers respond to threats to their identities. It is important to highlight that our literature review presents sensitising concepts (Bowen, 2006; Felix and Cavazotte, 2019) and is not meant to provide base theories for our field study. Grounded theory is constructed from an iterative process of waves of data construction and data analysis with the aim of building a theoretical model that is grounded on the data and that describes with parsimony the phenomenon that is being studied.

To select the participants for this study, we sought to invite individuals who worked at least 50% of the time remotely and who answered positively the question: “An identity is a definition that a person makes about who he or she is. Therefore, are you of the opinion that you have a remote worker identity?” For this, we sent an email inviting them to participate in the survey to a list of 9,413 alumni of a Brazilian Business School, which contained both questions. A total of 102 individuals responded positively to both questions.

Initially, we randomly interviewed 14 individuals from among the 102 possible respondents. In these interviews, we used an initial version of the interview protocol. Subsequently, we analysed the data according to the principles of grounded theory (Charmaz, 2014), which led us to create first-order codes. These codes were more descriptive in nature and were created with verbs in the gerund with the purpose of denoting the actions in the analysed passages (Felix et al., 2018). These initial codes led us to reformulate our interview protocol, and this new version was used for the second wave of data collection, in which we performed another 15 interviews with remote workers. After this new round of interviews, we performed a new wave of data analysis and revised the interview protocol. This iterative process of data construction and analysis and reformulation of the interview protocol took place sequentially, with another three rounds of immersion in the research field. In the third, fourth and fifth waves of data collection, we performed 11, 16, and 15 new interviews, respectively. The interval between each round of data collection was approximately 3 weeks. Thus, after completing all rounds of data collection and analysis, we conducted a total of 71 interviews, all of which were conducted in person. The fourth question of the final interview protocol, presented in Table 1, was included in the second wave of data collection, and the seventh question was added after the third round. For all interviews, we transcribed the segments that were subject to coding, resulting in a total of 348 pages of transcribed data. The criterion to define when to finish the data collection and analysis process was theoretical saturation (Charmaz, 2014), which means no new codes emerged in the final wave of data analysis.

**TABLE 1:** Interview protocol.

Sample questions
Tell me a little about your professional trajectory so far.
What are the personal values that have guided your career so far?
How long have you been working remotely?
On average, what percentage of hours do you spend working remotely?
Do you see yourself as a remote worker? Has always it been like that? How important is this identity to you?
Have you ever felt your worth being challenged or threatened at the company that you work for (or a company that you worked for in the past) because you work remotely? If so, what happened?
How did you react to these threats to your identity as a remote worker? Why do you think that you reacted this way? Did you still feel threatened after acting this way?

For analysing our data, we employed a two-step coding process similar to the one used by Kreiner et al. (2009). First, we developed inductive codes based on the data, considering each phrase and word as viable data units that were subject to coding (Heath and Cowley, 2004; Felix et al., 2023b). In this initial step, we recorded all first-order codes in an emerging codes dictionary. Then, two out of three authors independently analysed the interview excerpts supporting each code and tried to group them into the initially generated first-order codes, similar to what was done in previous studies (Mace and Ward, 2002; Stough and Lee, 2021). The coders were free to assign a given piece of data to one of the previously constructed codes or eliminate it. In cases of disagreement, an independent judge analysed the interview excerpt. This process created opportunities for theory development as the disagreements led us to reflective conversations about the data. This process was performed at the end of each wave of data collection, which allowed us to refine the code dictionary. So, our coding process was performed from multiple points of view, what reduces the potential for interpretation bias (Kolb, 2012).

**FIGURE 2:**
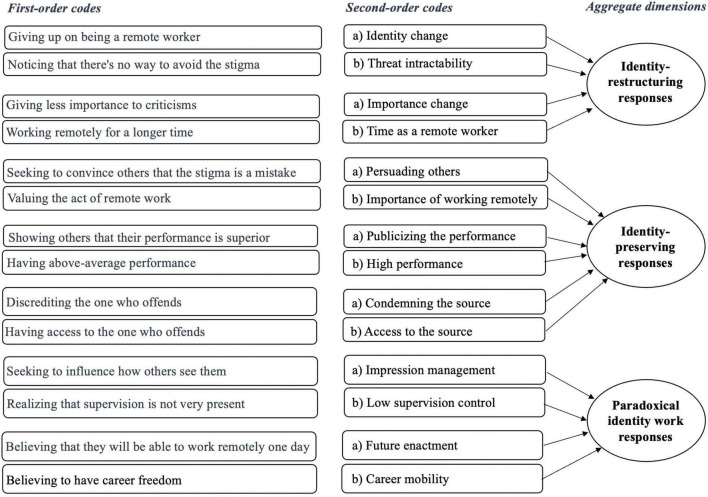
Data structure.

Once the first-order codes became more stable, we proceeded to analysis with the goal of grouping them into more abstract codes known as second-order codes. The same coding verification process, involving two authors and subsequent analysis by an independent judge, was applied in generating second-order codes from our list of first-order codes. Once the second-order codes also demonstrated greater stability, we replicated the same process to group them into aggregated dimensions, which represent even more abstract codes with broader theoretical significance. This process is also present in other grounded theories (Byron and Laurence, 2015; Felix and Cavazotte, 2019). The overall percentage of agreement between the two coders was 0.94, which exceeds the minimal 0.70 threshold suggested by Cohen (1960) and Kreiner et al. (2009). In Figure 2, we present the data structure resulting from the data analysis process, illustrating the path from first-order codes to aggregated dimensions.

The demography of the interviewees is characterised as follows. Their ages ranged from 22 to 62 years old, and 36 were males and 35 were females. Additionally, 48 of our research participants were married or were living with a significant other, 12 were in a committed relationship but living apart and 11 were single. The following sectors were represented in the study: technology (11), entertainment (9), banking (6), consumer products (6), advertising (6), manufacturing (6), education (6), chemicals (4), construction (4), utilities (3), hospitality (3), healthcare (3), mining (2), and transportation (2).

The analysis process followed grounded theory procedures. Initially, the first-order codes were grouped into second-order codes. Then, we analysed them and grouped them into aggregate dimensions. These steps of analysis led us to build codes with three different levels of abstraction. First-order codes are more descriptive and closer to the data, while second-order codes, and especially the aggregate dimensions, are more theoretical and analytical, as they show a higher degree of conceptual abstraction. We completed the process of constructing our model when we linked some of the codes and dimensions that showed a greater power of theoretical abstraction into a model and created ten propositions. In the following section, we present our results and the model that we created through the methodological process presented here.

## Results

**FIGURE 3:**
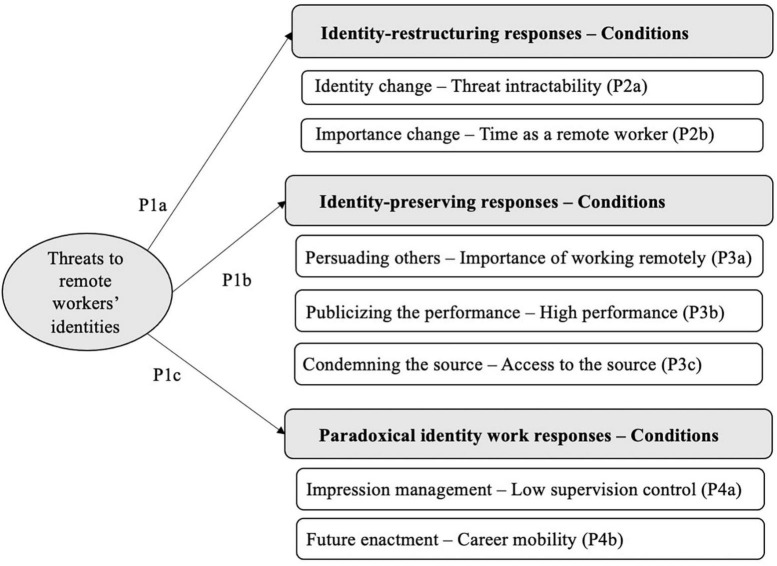
A model of responses to threats to remote workers’ identities.

In this section, we seek to understand the responses the remote workers adapt to threats to their identities (proposition 1). Then, we explore the conditions under which each response is more likely to occur (propositions 2a to 4b). Figure 3 introduces our grounded model, which we explain in more detail in the following sections.

### Remote workers’ responses to identify threats

Initially, we seek to understand what responses the remote workers interviewed present when their identities are threatened. Our data analysis process allowed the construction of three categories of responses, which we coded as “identity-restructuring coping responses,” “identity-preserving coping responses,” and “paradoxical identity work responses.” For each of these categories, some specific types of responses were found. Although the first two categories can be found in the literature on identity threats (Piening et al., 2020), we decided to include them because the specific responses that composed them were innovations of this study. Next, we present each of these categories, their definitions, and the empirical evidence that supports their constructions.

We coded as “identity-restructuring coping responses” the situations in which remote workers reported that, when faced with threats to their identity, they sought to alter aspects of their self-concept to better fit their social context. This category involved two specific types of responses: (1) disengaging from the remote worker identity (identity change) and (2) altering the salience of the remote worker identity in the individual’s own hierarchy of identities (importance change). In Table 2, we present this category, the specific types of responses, their descriptions, and a description of the conditions in which they are more likely to occur and a sample quote that highlights each type of response.

**TABLE 2:** Identity-restructuring coping responses.

Identity-restructuring coping response	Description	When is the coping response pursued?	Sample quote
Identity change	Disengaging from the remote worker identity	When the threat is regarded as intractable	“I gave up on being a remote worker; it was too much trouble. I tried a lot of things. I sent out media articles, blogs—showing that it works. I talked to people. I showed that the people complaining the most about remote work are the people who are producing less here. I saw that it was going to harm my career, so I gave up. I started working in the office. (…) After that, I didn’t feel that I was being judged anymore. I didn’t feel any threat” (38-year-old man, human resources analyst).
Importance change	Altering the importance of the remote worker identity in their own hierarchy of identities	When the identity as a remote worker has developed recently	“I still work remotely sometimes, but being competent and cooperative with the company’s philosophies became more important to me than being someone who works remotely. I remember that at first, I was proud to be building a career that allowed me to be a remote worker. But when I saw that the costs of doing this were really high, I started seeing [remote work] as less important and valuing other things more (…). I had been working like this for a short time, so it was not very complicated to change, to see myself again as someone who works face to face (…). This changed everything: I no longer feel that my self-esteem was attacked; I gave in; I changed for them” (29-year-old man, IT analyst).

“Identity change” refers to exiting the remote worker’s self-concept with the intention of eliminating the likelihood of potential harm. In the example cited in Table 2, the interviewee reports that he no longer sees himself as a remote worker, even though he continues to perform professional activities at home approximately 50% of the time. He and other research participants understood that this identity represented high risks for their self-esteem and, thus, they preferred to eliminate “being a remote worker” as a constituent element of their identities. In turn, “Importance change” refers to decreasing the motivational significance of being a remote worker and, consequently, the potential impact of the threat to their self-concept. In the example that we presented in Table 2, the interviewee started to define himself as “someone who works face to face,” even though he worked approximately 60% of his time remotely to reduce the negative effects of the stigma associated with not working in person at his company’s office.

The second group of responses was coded as “identity-preserving coping responses.” They refer to actions directed at the source of the threat and that involve maintaining the identity of the remote worker. This category encompasses three types of responses: (1) attempting to personally convince individuals who have a negative view of remote workers that they are productive (persuading others), (2) disclosing information in a depersonalised way that shows that working remotely makes it easier to improve productivity (publicising the performance), and (3) discrediting the one(s) who threatens the value of remote workers (condemning the source). In Table 3, we present the details of this category and an example of empirical evidence that supports it.

**TABLE 3:** Identity-preserving coping responses.

Identity-preserving coping response	Description	When is the response adopted?	Sample quote
Persuading others	Making personal attempts to convince individuals who view remote workers negatively that the latter are productive	When the remote worker identity is important to the threatened individual	“When I started feeling judged for being a remote worker, my reaction was to talk one on one with the people at the company. I didn’t want to give that up because I feel good that way, and I want to build a career working remotely. I want to live in several places in the world over the course of my life, and being a remote worker makes that possible. So I did a little ant job of convincing [people] one by one. (…) Because I was really dedicated, the result was that they saw that I’m not a lazy person who wants to stay at home, and today I don’t feel threatened anymore” (23-year-old woman, accounting analyst).
Publicising the performance	Disclosing information in a depersonalised way to show that working remotely makes improving productivity easier	When the performance as a remote worker is above average	“Since I started working remotely in the pandemic, I noticed and decided that I want to work like this my whole life. But, yes, I felt very judged after the pandemic ended when people wanted to come back and I didn’t. What I do to reduce this discrimination is to make the productivity of my team more explicit to people in general. I started putting team outcomes over the last month in my email signature that I use internally for other employees. I put a whiteboard in my workplace with my completed tasks; it is in a place that people can see it. Of course, my outcomes are much higher than the average of those who are working in person (…). I still feel threatened because I don’t know if they have changed their minds about me, but this was a way that I found to be more confident in continuing to working remotely” (47-year-old man, sales manager).
Condemning the source	Discrediting those who threaten the value of remote workers	When access to the source of the criticism is limited	“I have always seen myself as a remote worker. It was always good, but I felt that they saw me as someone who doesn’t work hard. (…) I felt threatened, yes. [At this company], I saw that they thought of me as a deceptive person. (…) What I do is, whenever I hear some criticism, I try to find out where the criticism came from and then show how that person is not that smart. Last week, I was told that our CEO in the United States said that working remotely was something that was necessary because of the pandemic and that anyone who wanted to continue working at home was uncommitted. I started showing people the mistakes in his management strategy; he has no basis to talk about creativity. (…) I think that I even convinced some people that he is wrong, but most people are still judging me. I feel that. I wouldn’t do that if the criticism came from my immediate superior. The fact that he is far from me makes me more comfortable in criticising him” (35-year-old woman, customer service supervisor).

Among the identity-preserving responses, “persuading others” tends to occur when there is an attempt to convince other people that working remotely is not indicative of less dedication or productivity at work. “Publicising the performance,” in turn, describes situations in which the threatened remote worker seeks to publicly display his or her results and/or those of the team. As can be identified in the example shown in Table 3, this display is done without focussing on any specific target. Finally, “condemning the source” refers to situations in which the remote worker criticises the professional competence of the individual(s) who are characterised as the source(s) of the threat, such as a CEO or a public figure.

The third and final category was named “paradoxical identity responses.” It refers to ambiguous and non-mutually exclusive actions between preserving and restructuring the remote worker identity. This category encompasses two types of responses: (1) maintaining the remote worker identity in one’s self-concept, but seeking to make significant others disassociate him or her from this identity (impression management), and (2) giving up the remote worker identity in the present and keeping an expectation of enacting it in the future (future enactment). In Table 4, we present this category, its two specific types of responses, the descriptions, a description of the conditions in which they are more likely to occur and a sample quote that highlights each response.

**TABLE 4:** Paradoxical identity work responses.

Paradoxical identity work response	Description	When is the coping response pursued?	Sample quote
**Impression management**	Maintaining the remote worker identity in one’s self-concept but seeking to make significant others disassociate him/her from this identity	When supervision control is low	“Since I started to feel challenged for being someone who prefers to work from home, I’ve started to kind of manage other people’s interpretations of me. Sometimes I go [in to work], and I have lunch there. I spend some time in the cafeteria because I want to be seen. If I’m in my office and I hear my director’s voice, I go into the hallway, pretend I’m going to get a coffee, and then go home. So I still see myself as a remote worker, but I don’t feel it at the same time because I always try to signal that I am often at the office. (…) Because I know my boss doesn’t monitor us closely, whether we are there or not. If he were a more physically present person, this wouldn’t work. I think that this tactic lessens the threat, but it won’t work for a long time. I know that” (53-year-old man, product manager).
**Future identification**	Giving up the remote worker identity in the present and keeping an expectation of identifying that way in the future	When the remote worker’s career mobility is high	“I do feel like a remote worker, but I started working only half of the time at home when I noticed that I was being criticising for spending so much time working from home. In practical terms, I don’t know if I see myself as a remote worker anymore because I’ve become a hybrid. But I know that in my next job, I will certainly choose a place that sees remote work as normal, as something positive. I know that I can get this king of a job (…). When I think like this, the threat to me as a remote worker doesn’t affect me anymore. I’ll end up leaving this company, and while I’m here, it’s going to be progressively harder to hide my dissatisfaction” (28-year-old woman, programmer).

“Impression management” refers to trying to make other people not interpret him or her as remote workers, while the individual, internally, maintains this characteristic as a component of his or her self-concept. In the case presented in Table 4, the interviewee reports that he began to “take care of appearances” and “be seen” in a way that lessens the threat to himself. “Future enactment” describes how the threatened remote worker understands that it will not be possible to enact this identity in the present but maintains this element as a constituent of an alternate future self-concept. In the empirical evidence presented, the research participant says that she no longer sees herself as a remote worker at present but that she will certainly do so in her next job. Thus, she performs a future enactment of her remote worker identity, keeping it as part of her self-concept.

Thus, in view of the evidence presented, we theorise that:

**Proposition 1:** Individuals respond to threats to their remote worker identity by adopting (a) identity restructuring (“identity change” and “importance change”); (b) identity-preserving (“persuading others,” “publicising the performance” and “condemning the source”); or (c) paradoxical identity work (“impression management” and “future enactment”) responses.

### Conditions influencing the pursuit of each identity-restructuring response

To answer our second research objective, we sought to understand the conditions under which each coping response tends to occur more frequently, starting with the identity-restructuring responses. The adoption of the “identity change” response is a costly way of responding to a threat, since it involves disengagement psychologically and physically from what sustains the self-concept of being a remote worker. Therefore, several interviewees said that they avoided adopting this answer as much as possible. However, in some cases, participants reported that they “had no way out,” that “it became impossible” or that they were “cornered.” We coded cases such as these and what we present in Table 2 as “threat intractability,” that is, situations in which the individual understands that the damage caused if he or she decided to keep his or her threatened identity would be irreversible. In cases where remote workers reported that they interpreted the threat as intractable, the option for identity change was more frequent. Thus, we suggest the following:

**Proposition 2a:** When faced with the identity threats that provoke identity-restructuring responses, the stronger the intractability of the threat to one’s remote worker identity, the more likely individuals will pursue an identity change.

Not all threats to remote workers’ identities are intractable. We noticed that, for some respondents, the identity as a remote worker was negotiable, depending on the costs that would be required to enact it. Especially in the case of remote workers who had recently adopted such an identity, the relativisation of this identity was recurrent. In the case of the interviewee shown in Table 2, he said that he began to “give less importance to this (being a remote worker) and value other things,” as he realised that such an identity was threatened and it would be very costly to enact it in the present. Thus, we propose the following:

**Proposition 2b:** Among identity threats that provoke identity-restructuring responses, the more recent the identity as a remote worker, the more likely individuals will pursue an important change.

### Conditions influencing the pursuit of each identity-preserving response

Next, we seek to understand the conditions that lead remote workers to maintain their identities, even when threatened. For some respondents, being a remote worker is a high priority in their identity hierarchy. In these cases, the interviewees reported that after having their remote worker identity threatened, they tended to seek to “persuade others” about an alleged mistake in judgements involving the threat. In the empirical evidence presented in Table 3, the interviewee says that she did a “little ant job, of convincing people one by one.” As several other cases showed the same pattern, we theorise the following:

**Proposition 3a:** Among identity threats that provoke paradoxical identity work responses, the stronger the importance of working remotely, the more likely that individuals will pursue a strategy of “persuading others.”

On the other hand, some interviewees reported that they were able to obtain more positive performances than those presented by individuals who work non-remotely. In these cases, several participants reported that they started to find ways to “publicise the performance” and show, in an impersonal way, evidence that the threats to their remote workers’ identity were not grounded in reality. In the case of the interviewee shown in Table 3, the strategies used were to report his performance in the email signature and on a whiteboard easily accessed visually by his colleagues. Thus, we suggest the following:

**Proposition 3b:** When faced with identity threats that provoke paradoxical identity work responses, the better the individual’s performance, the more likely the individual will pursue “publicising the performance.”

We also identified that, in some cases, the threat to the remote workers’ identity occurred not from a colleague with whom they had daily contact but from a leader or public person with whom the individual had little or no contact. In cases where there was less access to the source of the threat, we noticed that the most common response was “condemning the source,” that is, to carry out an ad hominem attack on the individual who carried out the stigmatisation. In the case of the interviewee shown in Table 3, the criticism against remote workers came from the CEO of the company where she works. Since this CEO works in another country and there is no personal contact with him, the interviewee reported that she feels more comfortable seeking to invalidate his criticism by exposing his “management mistakes.” In our analysis, we found similar reports regarding world-renowned leaders, such as the aforementioned Elon Musk. This led us to propose the following:

**Proposition 3c:** When faced with identity threats that provoke identity-preserving responses, the less access to the source of the threat, the more likely that the individuals will pursue “condemning the source.”

### Conditions influencing the pursuit of each paradoxical identity work response

Finally, we also seek to understand the conditions under which paradoxical identity work responses tend to be adopted. The response “impression management” was more common when respondents reported that they had less supervisor control. While some remote workers reported that their leaders have clear indicators of their performance, as well as control mechanisms to assess whether they are actually working at the expected time, others reported the opposite. When the scenario was one of little supervision, several research participants reported that they felt the need not only to obtain satisfactory results but also to influence the perception of their leaders in everyday situations. These micro impressions involved, for example, artificially provoking situations such as visiting the office and being seen by their leaders, as indicated in Table 4. Therefore, we suggest the following:

**Proposition 4a:** When faced with identity threats that provoke paradoxical identity work responses, the less the supervision, the more likely that individuals will pursue “impression management.”

The second paradoxical identity work response that we found was “future enactment.” According to our analysis, this response was more common in cases where remote workers understood that they have high career mobility and, consequently, alternate professional selves in which remote work is well regarded. More specifically, this response was recurrent when respondents reported that they would not be able to access an alternative professional identity that involves remote work in the short term. Thus, they minimised their frustrations of being stigmatised by anchoring themselves in a future expectation of enacting their remote work identity. The interviewee’s report that we present in Table 4 shows the paradoxical nature of this type of answer: she says that in practice, she no longer sees herself as a remote worker, but she also says that she still sees herself as such, since her next job will certainly involve remote work. In this way, we theorise that:

**Proposition 4b:** When faced with identity threats that provoke paradoxical identity work responses, the greater the career mobility, the more likely that the individuals will pursue “future enactment.”

## Discussion

Our intent in this paper was to understand (a) how remote workers respond to threats to their identity and (b) the conditions under which each coping response tends to occur more frequently. Using the grounded theory approach, we found answers to our two research objectives. A relevant aspect in which the study advances the literature on remote work involves the negative stigmas attributed to remote workers (Bornstein, 2013; Barhate and Hirudayaraj, 2021) and the lack of understanding of how they respond to those threats in existing studies. In this study, we showed that the answers presented by these workers are not homogeneous and that they have implications for the continuity of the remote workers’ identity. The identity lens used in this study (Tajfel and Turner, 1979) also allowed us to identify that the self-concept of a remote worker does not necessarily depend on whether the individual is acting as such. Coping responses such as “impression management” and “future enactment” illustrate this theoretically relevant idea, since common sense and previous studies suggest that identities would be associated with an enactment that should be grounded in real action (Felix and Cavazotte, 2019).

Previous studies have also suggested that remote workers’ lower organisational visibility makes them need to manage other people’s impressions of them (Barsness et al., 2005; Shockley et al., 2021), as they are less easily “seen” (Barsness et al., 2005; Bailey and Kurland, 2002; Bolduan et al., 2021; Fang et al., 2022). In this study, we showed that when the identity threat is seen as intractable or when the remote worker identity is recently acquired, the costs for maintaining this self-concept become so high that the individual tends to opt for an identity exit. However, when the remote worker’s identity is considered to be of high importance, when the individual’s performance is superior, and when the source of the threat is distant, the worker tends to continue to see him or herself as a remote worker. We also show that conditions such as low supervisor control and the expectation of working in a future job remotely leads individuals to adopt paradoxical responses. These results allow an advance to the specific literature on the stigmatisation of these workers (Bornstein, 2013), as they expand the understanding of “if” they are stigmatised, to “how” they respond to identify threats and under what conditions.

Furthermore, previous studies on remote work discuss the impacts that the negative stigma directed at these workers generates for their careers (Chung, 2020; Padavic et al., 2020; Becker et al., 2022; de Laat, 2022). In this study, we theorised about how and about the circumstances under which their careers tend to be impacted. We show that many remote workers who see themselves as productive fail to adopt this form of work merely as a defencive response in the face of stereotyped social judgements. Thus, this study shows the need to shed light on this phenomenon, given that, in a world impacted by the COVID-19 pandemic, an increasing number of individuals will resort to remote work. Our model shows the risks to remote workers of redesigning their careers due to the stigma attached to a work modality necessary for the current work context.

We add to the existing meso and micro approaches to remote work (Lin, 2011; Adamovic, 2018; Mockaitis et al., 2018; Donnelly and Johns, 2021; Williams et al., 2021) by focussing on the micro-interactions in which remote workers engage or which they consciously create to manage threats to their remote workers identity, thus highlighting how the remote workers themselves have the agency to “deal with” and manage identity threats. By reflecting upon remote workers’ identity threats, and their micro-interactive responses to it, in light of the wider macro context—in this case: the political framing of remote workers’ identities in Brazil—we show the relevance of analysing how micro-interactions and macro-level boundary conditions inform each other.

## Conclusion

Our model and theoretical propositions create insights into how remote workers respond to negative stigma from a range of origins. While some responses lead to restructuring the remote workers’ identity (identity restructuring responses), others involve keeping the enactment of such identity (identity-preserving responses) or maintaining a paradoxical relationship between restructuring and preserving the identity (paradoxical identity work responses). We also theorise on the conditions under which each response is more likely to occur. Next, we present the limitations and avenues for future research and also discuss how our results can unfold into practical actions.

### Limitations and future research

This study has limitations, which can be addressed in future studies. First, although we found several responses to remote workers’ identity threats, for questions of parsimony, we did not explore the outcomes of those responses. Future studies could explore which of them maintain or eliminate the threat both in the short and long term. Second, we explored the experience of the threatened individual, but we did not seek to understand the impacts of each response in terms of work-related outcomes. What are the costs of “impression management” in terms of productivity? What are the consequences of “persuading others” in terms of the level of stigma toward the threatened individual? Third, we have only adopted an individual-level perspective on identities. Future studies could explore how other identity levels of analysis, such as the group and organisational levels, interrelate and influence how remote workers respond to threats to their identities. Given the specifics of the Brazilian context—that is: the stigma attached to remote work on the level of politics—it would be highly relevant to conduct comparative studies that take these macro-level influences into account.

### Practical implications

This study has implications for practice. For remote workers, we provide a list of responses that can help individuals protect their sense of self when stigmatised for working remotely. Identity threats represent a risk to the career of individuals, and the study of coping responses becomes essential for individuals to manage their career development in non-ideal environmental conditions. More specifically, we suggest that individuals who have work preferences evaluate how willing they are to take the risk of reinforcing their identities and advocating for their preferred way of working, or adapting to environmental expectations. However, our model has shown that the responses do not need to be binary or mutually exclusive: through paradoxical responses, individuals can, at least temporarily, meet their needs for authenticity while also conforming to the need to be accepted. In a world where it is not always possible to be completely authentic, sometimes being “authentic enough” (Hennekam and Ladge, 2022; Felix et al., 2023e) can be a more viable path.

For human resource management (HRM) professionals, the study highlights the importance of raising awareness among managers about the consequences that stigma toward remote workers may have on the performance of these individuals and of the organisation. Given that the choice to offer the possibility of working remotely is up to the organisation, once such a choice is made, it makes sense that the organisation seeks to provide the necessary conditions to maintain the self-esteem and performance of the employee. However, in cases where organisations are led by professionals who have a negative view of working remotely, as in the case of Elon Musk, the possibilities for action of HRM professionals become limited. Our data shows that HRM professionals should pay special attention to the process of raising awareness among top leadership, as reports indicate that a significant portion of actions considered identity threats by employees originate from high-ranking leaders.

We also contribute to a key International HRM challenge, namely the need to consider both universal and local factors when designing and implementing HRM actions. For managing remote workers’ identities, this implies the need to look beyond the organisational meso-context, to also view micro-level identity processes in light of their macro-boundaries and to consciously position the organisation, and its HRM response, as a buffer, mediator or interlocutor between macro and micro. Otherwise, it is likely that the organisation will end up adopting universal standards regarding the location where work should take place, which may lead to lower productivity in certain regions of the world.

## Data availability statement

The raw data supporting the conclusions of this article will be made available by the authors, without undue reservation.

## Ethics statement

The studies involving humans were approved by the Fucape Business School Ethics Committee. The studies were conducted in accordance with the local legislation and institutional requirements. The participants provided their written informed consent to participate in this study.

## Author contributions

BF and BT conceived of the presented idea, collected and analysed the data, and wrote the manuscript. JM and RD verified the analytical methods, discussed the results, and contributed to the final manuscript. All authors contributed to the article and approved the submitted version.
